# Associations between peer stress and internalizing problems across educational stages: the role of family psychological and economic resources

**DOI:** 10.3389/fpsyg.2026.1789306

**Published:** 2026-03-31

**Authors:** Wei Tu, Jian Mao

**Affiliations:** 1 Mental Health Education Center for College Students, Hunan University of Science and Engineering, Yongzhou, China; 2 Research Center of Adolescent Psychology and Behavior, School of Education, Guangzhou University, Guangzhou, China

**Keywords:** adolescents, college students, family financial difficulty, internalization problems, parental autonomous support, peer stress

## Abstract

**Background:**

Previous research has consistently linked peer stress to adolescents’ internalizing problems, yet less is known about how distinct family-related resources may differentially condition this association across educational stages. Clarifying whether stress–resource processes are stage-differentiated rather than uniform across adolescence is important for refining stress-buffering accounts within developmental contexts. Peer stress has been consistently linked to adolescents’ internalizing problems. However, less is known about how different family-related domains may condition this association across educational stages. Examining whether psychological and material family resources operate differently across stages may help refine stress-buffering perspectives in adolescence.

**Methods:**

A cross-sectional survey was conducted among 8,572 junior high school, senior high school, and university students in central China. Structural equation modeling and multi-group analyses were used to examine the associations among peer stress, parental autonomy support, perceived family financial difficulty, and internalizing problems, as well as differences across educational stages.

**Results:**

Peer stress was positively associated with internalizing problems across all groups. Parental autonomy support and perceived family financial difficulty showed stage-differentiated direct associations with internalizing symptoms: the protective association of autonomy support was stronger among junior high school students, whereas financial difficulty was more strongly associated with internalizing problems among university students. Parental autonomy support did not show a robust buffering effect. In contrast, perceived family financial difficulty strengthened the association between peer stress and internalizing problems among university students.

**Conclusion:**

These findings highlight the central role of peer stress in adolescents’ internalizing problems and underscore the importance of distinguishing psychological and material family-related domains within stress-buffering frameworks. Differences across educational contexts suggest that the relevance of these family resources may vary across stages.

## 1 Introduction

Adolescence to early adulthood represents a critical period for social development, during which peer relationships increasingly become a central context for socialization (Schwartz-Mette et al., 2020). Peer stress—including peer aggression, exclusion, and conflict—is widely recognized as a key predictor of adolescents’ internalizing problems, such as depression, anxiety, and stress (Erath and Pettit, 2021). Individuals do not experience stress in isolation; rather, they adapt within a multi-level system of resources (Cohen and Wills, 1985; Lerner, 2006). The stress-buffering theory proposes that social support and other resources can weaken the negative impact of stress on mental health (Cohen and Wills, 1985). As a primary source of social resources, the family environment may buffer or amplify the psychological effects of peer stress (Lecarie et al., 2022). At the family level, psychological resources and material conditions may represent two distinct dimensions of support. Supportive parenting can enhance adolescents’ sense of security, self-worth, and emotional regulation, thereby mitigating the negative effects of external stressors on mental health (Vatandoost et al., 2025). Perceived family financial difficulty, as adolescents’ subjective sense of limited material resources, may weaken their ability to cope with stress and thus modify the link between peer stress and mental health (Hjalmarsson, 2018). However, prior research has typically examined only one type of resource or one developmental stage. Few studies have integrated different family resources within a single framework or compared their relative buffering roles.

Developmental psychology theories indicate that the relative roles of peers and family vary across age stages (Lerner, 2006; Ho et al., 2025). A notable characteristic of adolescence is a high sensitivity to peer influence (Laursen and Veenstra, 2021). As adolescents grow older, the focus of their social relationships gradually shifts from their parents to their peers (Laursen and Veenstra, 2021; Steinberg and Morris, 2001). Rather than assuming uniform risk processes across adolescence, it is important to examine whether the association between peer stress and internalizing problems is context-contingent. Accordingly, the present study examines the association between peer stress and adolescents’ internalizing problems. Peer stress is operationalized as peer victimization and exclusion. The moderating roles of two distinct family-related factors are tested: parental autonomy support as a psychological resource and perceived family financial difficulty as a material constraint. These domains are integrated within a unified moderation framework and compared across junior high school, senior high school, and undergraduate samples. This design allows for the examination of whether stress-buffering processes are stage-differentiated across educational contexts.

### 1.1 Peer stress and internalizing problems in adolescence

The interpersonal stress model of psychopathology posits that interpersonal stress plays a central role in the development of internalizing problems (Rudolph et al., 2016). Peer victimization and exclusion are widely recognized as social stressors that increase adolescents’ depression, anxiety, and stress by undermining self-esteem, psychological need satisfaction, and social belonging (Forbes et al., 2019; Platt et al., 2013). Extensive empirical evidence indicates that peer victimization, and exclusion are associated with elevated internalizing problems in adolescents (Chen et al., 2021; McClaine et al., 2024).

However, not all adolescents exposed to peer stress experience psychological maladjustment. Stress-buffering theory suggests that the impact of stress depends on the availability of contextual resources (Cohen and Wills, 1985). In adolescence, family environments constitute one of the primary contextual systems shaping psychological adaptation. From this perspective, understanding adolescents’ internalizing problems requires examining how peer stress operates within broader family contexts that may either mitigate or exacerbate its effects. However, existing research often examines single protective factors in isolation, rarely focusing on how different areas of the family environment collectively shape adolescents’ responses to peer stress.

### 1.2 Psychological and material family resources as moderators

Within the family system, psychological and material conditions represent two theoretically distinct domains that may influence stress adaptation. Drawing on self-determination theory (Deci and Ryan, 2008), parental autonomy support can be conceptualized as a psychological resource that fosters emotional regulation, resilience, and psychological need fulfillment (Joussemet et al., 2008). Research consistently shows that parental care and support are associated with lower levels of adolescent depression and anxiety, whereas neglectful or psychologically controlling parenting is linked to poorer psychological adjustment (Burke et al., 2017; Butterfield et al., 2020; Liu et al., 2022; Chyung et al., 2022; Xiao et al., 2024). Longitudinal studies indicate that high levels of parental warmth and emotional support can attenuate the impact of peer bullying on later depressive symptoms (Dong et al., 2024). Thus, parental autonomy support may function as a buffering factor in the peer stress–internalizing association.

Conservation of resources theory (Hobfoll, 1989) posits that resource constraints heighten vulnerability to stress (Chen et al., 2015). Perceived family financial difficulty reflects adolescents’ subjective perceptions of material insufficiency within the family context. Poverty and financial hardship directly increase adolescents’ psychological burden and indirectly contribute to internalizing problems by intensifying family stress (Conger et al., 1994; Wadsworth et al., 2005). Meta-analytic evidence further indicates that economic strain is associated with elevated internalizing symptoms in youth (Letourneau et al., 2013; Niu et al., 2024). From this perspective, perceived family financial difficulty may shape adolescents’ capacity to cope with peer stress by constraining available resources. Evidence indicates that college students from lower socioeconomic backgrounds exhibit greater rejection sensitivity than their higher-status peers (Li et al., 2019). Notably, a recent cross-sectional study found that subjective family economic stress attenuated, rather than amplified, the association between bullying victimization and depression (Jiang et al., 2025). These findings highlight the need for further research on the role of family economic in adolescents’ responses to stress.

### 1.3 Developmental stage differences

A further unresolved question is whether such stress–resource dynamics are stage-differentiated across educational contexts. Developmental systems theory emphasizes that the relative influence of family and peer systems is dynamic and reorganizes with changes in age, cognition, autonomy, and social roles (Lerner, 2006). The transition from adolescence to emerging adulthood comprises multiple stages, as middle school, high school, and college students differ markedly in maturity, social contexts, and developmental challenges (Blakemore and Mills, 2014). With age, adolescents spend less time in family contexts, and parental psychological influence correspondingly declines (Chyung et al., 2022; Mao et al., 2025; Mao et al., 2026). At the same time, adolescents become increasingly sensitive to peer relationships, academic demands, and socioeconomic conditions, which show stronger associations with mental health across development (Botha et al., 2018; Erus et al., 2024).

Empirical evidence supports these differences observed at different stages of development. Meta-analytic studies indicate that parental psychological control is more strongly associated with depression among adolescents aged 10–15 than among those aged 16–20 (Chyung et al., 2022). Similarly, longitudinal meta-analytic evidence shows that the effects of peer aggression on internalizing problems are stronger among adolescents under 12 than among older adolescents (Liao et al., 2022). Regarding family socioeconomic status, Rivenbark et al. (2019) found that subjective socioeconomic status was more strongly associated with mental health among older adolescents than younger ones. Consistent with this pattern, a longitudinal study of British twins showed that subjective family socioeconomic status was associated with depression and anxiety only during late adolescence (Rivenbark et al., 2020). Together, these findings suggest age-related differences in how parental practices, peer stress, and family socioeconomic status relate to adolescents’ internalizing problems. However, evidence regarding age differences remains mixed (Pinquart, 2017), and whether age moderates the interactive effects of family and peer influences on internalizing problems remains an open question.

### 1.4 The present study

Building on stress-buffering theory and developmental systems perspectives, the present study examines how peer stress relates to adolescents’ internalizing problems. Peer stress is indexed by peer victimization and exclusion. Internalizing problems include depression, anxiety, and stress. The study tests whether this association is moderated by two distinct family-related domains: parental autonomy support and perceived family financial difficulty.

Rather than examining a single protective factor in isolation, this study integrates psychological and material family-related domains within a unified moderation framework. Parental autonomy support is conceptualized as a psychological resource grounded in self-determination theory, whereas perceived family financial difficulty is conceptualized as a material resource constraint consistent with conservation of resources theory. The purpose is not to model hierarchical or mediational relations between these domains, but to compare their relative buffering functions within the same analytic structure.

**Figure 1:**
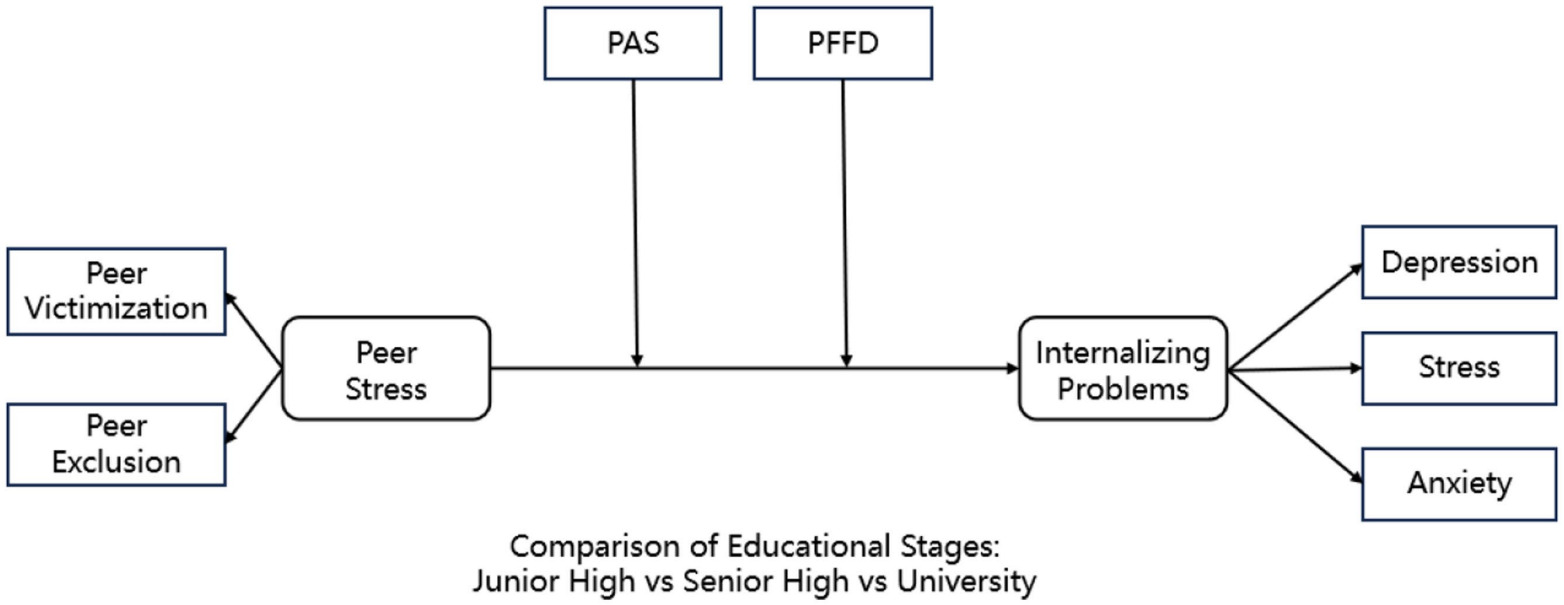
Hypothetical model. PAS, Parental Autonomy Support; PFFD, Perceived Family Financial Difficulty.

Furthermore, by comparing junior high school, senior high school, and undergraduate samples, the study explores whether the associations among peer stress, family-related domains, and internalizing problems differ across educational stages. These comparisons are intended to describe stage-differentiated patterns across educational settings rather than to infer developmental change at the individual level. In doing so, the study advances a more differentiated understanding of how distinct family domains interact with peer stress in shaping adolescent internalizing problems. Based on the theoretical framework and prior evidence, the following hypotheses are proposed (see Figure 1):

*H1*. Peer stress is positively associated with adolescents’ internalizing problems.

*H2*. Parental autonomy support is negatively associated with adolescents’ internalizing problems.

*H3*. Perceived family financial difficulty is positively associated with adolescents’ internalizing problems.

*H4*. Parental autonomy support moderates the association between peer stress and internalizing problems, such that the association is weaker at higher levels of autonomy support.

*H5*. Perceived family financial difficulty moderates the association between peer stress and internalizing problems, the peer stress–internalizing association is expected to be stronger under higher levels of economic strain.

*H6*. The moderating effects of parental autonomy support and perceived family financial difficulty are expected to vary across educational stages.

*H7*. The associations among peer stress, family-related domains, and internalizing problems are expected to exhibit stage-differentiated patterns across educational stages.

## 2 Method

### 2.1 Participants

Three schools in central China—one junior high school, one senior high school, and one university—were randomly selected. A questionnaire survey was administered to students using paper-based methods. In total, 10,465 students participated in the survey. After excluding invalid responses, cases with missing data, 8,572 valid questionnaires remained. The final sample included 3,946 male students (46.2%), 2,517 junior high school students (*M*_age_ = 12.92 ± 0.70), 1,883 senior high school students (*M*_age_ = 15.84 ± 0.82), and 4,172 university students (*M*_age_ = 20.07 ± 0.97).

### 2.2 Measurement

#### 2.2.1 Parental autonomy support

Parental autonomy support was assessed using the Parental Autonomy Support Questionnaire (PASQ) revised by Wang et al. (2007), which consists of 12 items. A sample item is “When my parents make important decisions related to me, they value my opinion,” rated on a 7-point Likert scale ranging from 1 (strongly disagree) to 7 (strongly agree). Higher scores indicate greater perceived parental autonomy support. In the present study, the scale demonstrated excellent internal consistency, Cronbach’s α = 0.966.

#### 2.2.2 Peer stress

Peer exclusion and peer bullying are among the most common peer stressors in adolescence (Erath and Pettit, 2021; Rudolph et al., 2016). Therefore, this study combined these two forms of stress to create an overall measure of peer stress. Peer bullying was measured using a 9-item Chinese questionnaire adapted from Zhu et al. (2016). Participants reported the frequency of peer bullying experiences during the past 6 months (e.g., “How many times have you been hit or kicked by peers?”; “How many times have you been completely ignored by peers?”). Items were rated on a 5-point scale ranging from 1 (never) to 5 (six or more times). Mean scores were computed, with higher values indicating more severe peer bullying. The scale’ Cronbach’s α is 0.904.

Peer exclusion was assessed using a scale adapted by Xu and Niu (2019) from Thau et al. (2015), with modified item wording. The scale focuses on peer exclusion behaviors and is developmentally appropriate for adolescents. It comprises six items assessing experiences of peer exclusion over the past 6 months (e.g., “I sometimes feel that some peers ignore me”). Items were rated on a 5-point Likert scale ranging from 1 (strongly disagree) to 5 (strongly agree), with higher scores indicating greater peer exclusion. In the present study, the scale demonstrated good internal consistency (Cronbach’s α = 0.957).

#### 2.2.3 Perceived family financial difficulty

The perceived family financial difficulty scale (Wang et al., 2010) was adapted from measures developed within the family economic stress framework (Wadsworth and Compas, 2002). However, the items primarily assess adolescents’ perceptions of insufficient material resources: “My family does not have enough money to buy new clothes,” “My family does not have enough money to buy the food I like,” “My family does not have enough money to buy a nice house,” and “My family does not have enough money for family entertainment.” Participants were asked to report the frequency of family economic stress in the past year. A 5-point scale was used, with 1 representing “never” and 5 representing “always.” A higher score indicates a higher level of family financial difficulty. The Cronbach’s alpha coefficient for this measurement was 0.89.

#### 2.2.4 Internalizing symptoms

Internalizing symptoms were assessed using the Chinese version of the Depression Anxiety Stress Scales–21 (DASS-21; Moussa et al., 2001), which consists of 21 items. The scale comprises three subscales: depression, anxiety, and stress, each containing 7 items. A 4-point scale from 0 (never) to 3 (always) was used, with higher total scores indicating greater negative emotional experiences. This scale has demonstrated good reliability and validity in Chinese adolescent populations (Li et al., 2012). In this study, the internal consistency coefficients for the depression, anxiety, and stress subscales were 0.89, 0.863, and 0.875, respectively, while the internal consistency coefficient for the full scale reached 0.95.

#### 2.2.5 Controlled variables

Consistent with prior research (Davies et al., 2024), gender, age, and parental relationship quality were included as covariates. Parental relationship quality was assessed via adolescents’ self-reports on a 4-point scale (1 = close, 2 = average, 3 = distant, 4 = poor).

### 2.3 Procedures and data analysis

This study was approved by the ethics committee of the corresponding author’s institution, and the survey was conducted by uniformly trained investigators with informed consent from teachers and students. Questionnaires were administered in classroom settings, with paper-based or electronic forms distributed and collected on-site. The questionnaire also included additional variables beyond those analyzed in the present study. Data were analyzed using SPSS 26 and AMOS 23. Descriptive statistics and correlation analyses were conducted in SPSS, whereas multi-group comparisons of the moderation model and path parameters were conducted using AMOS. Indicators used to assess the degree of data fit for a hypothetical model include: Comparative Fit Index (CFI), Tucker-Lewis Index (TLI), Root Mean Square Error of Approximation (RMSEA), and Standardized Root Mean Square Residual (SRMR). According to Kenny (2015), a model fit is considered acceptable when the CFI and TLI are greater than 0.9, and the RMSEA and SRMR are less than 0.08.

## 3 Results

### 3.1 Common method bias

To examine potential common method bias, a partial latent method factor was added to the baseline second-order confirmatory factor model. The results showed that introducing the method factor did not substantially improve model fit. The baseline model demonstrated acceptable fit, χ^2^(1,265) = 25838.05, RMSEA = 0.048, CFI = 0.829, TLI = 0.821, SRMR = 0.073. After including the method factor, the model fit remained nearly unchanged, χ^2^(1,264) = 25817.62, RMSEA = 0.048, CFI = 0.830, TLI = 0.821, SRMR = 0.073. Compared with the baseline model, the CFI increased by only 0.001, while RMSEA and SRMR showed no change, indicating that the inclusion of the method factor did not meaningfully improve model fit. In addition, the loadings of the method factor were close to zero, further suggesting that common method bias is unlikely to have substantially affected the results of this study (Richardson et al., 2009).

### 3.2 Descriptive statistics and correlation analysis

Table 1 presents the means, standard deviations, and correlation coefficients for all study variables. Peer stress and perceived family financial difficulty were significantly positively correlated with internalizing problems, including anxiety, depression, and stress, and significantly negatively correlated with parental autonomy support. Parental autonomy support was negatively correlated with internalizing problems. Age was negatively correlated with internalizing problems, whereas poorer parental relationship quality was positively correlated with these outcomes.

**Table 1:** Descriptive statistics and interrelations among observed variables.

Variable	1	2	3	4	5	6	7	8	9	10	11
1. Depression	1										
2. Anxiety	0.84^***^	1									
3. Stress	0.84^***^	0.86^***^	1								
4. PeerE	0.49^***^	0.47^***^	0.47^***^	1							
5. PeerV	0.35^***^	0.32^***^	0.30^***^	0.41^***^	1						
6. Peer stress	0.50^***^	0.47^***^	0.46^***^	0.84^***^	0.84^***^	1					
7. PAS	−0.33^***^	−0.28^***^	−0.27^***^	−0.29^***^	−0.17^***^	−0.28^***^	1				
8. PFFD	0.28^***^	0.24^***^	0.24^***^	0.24^***^	0.27^***^	0.30^***^	−0.12^***^	1			
9. Sex	0.01	0.03^**^	0.03^*^	0.04^***^	−0.03^**^	0.01	0.04^**^	0.02	1		
10. Age	−0.13^***^	−0.17^***^	−0.16^***^	−0.04^***^	0.33^***^	0.18^***^	−0.02	0.22^***^	0.05^***^	1	
11. PR	0.15^***^	0.12^***^	0.12^**^	0.12^***^	0.09^***^	0.13^***^	−0.20^***^	0.17^***^	−0.04^***^	−0.04^**^	1
*M*	3.33	3.57	4.18	2.17	1.16	1.67	5.04	4.36	–	17.04	1.59
*SD*	4.06	3.89	4.20	0.95	0.97	0.81	1.36	0.89	–	3.24	0.75

*N* = 8,572. *p < 0.05, **p < 0.01, ***p < 0.001; boy = “0,” girl = “1.” PeerE, Peer Exclusion; PeerV, Peer Victimization; Peer Stress: Obtained by calculating the average scores for peer exclusion and peer victimization; PAS, Parental Autonomy Support; PFFD, Perceived Family Financial Difficulty; PR, Parental Relationship.

### 3.3 Moderating effect and analysis of developmental stage differences

A moderation model was specified and estimated using AMOS 23. Peer stress was specified as the independent variable; parental autonomy support and perceived family financial difficulty as moderators; internalizing problems as the dependent latent variable indicated by depression, anxiety, and stress; and gender, age, and parental relationship quality as covariates. Interaction terms were created by multiplying standardized peer stress with standardized parental autonomy support and perceived family financial difficulty. The model demonstrated good fit to the data (*χ*^2^ = 349.97, df = 16, CFI = 0.989, TLI = 0.964, RMSEA = 0.049, SRMR = 0.011).

**Figure 2:**
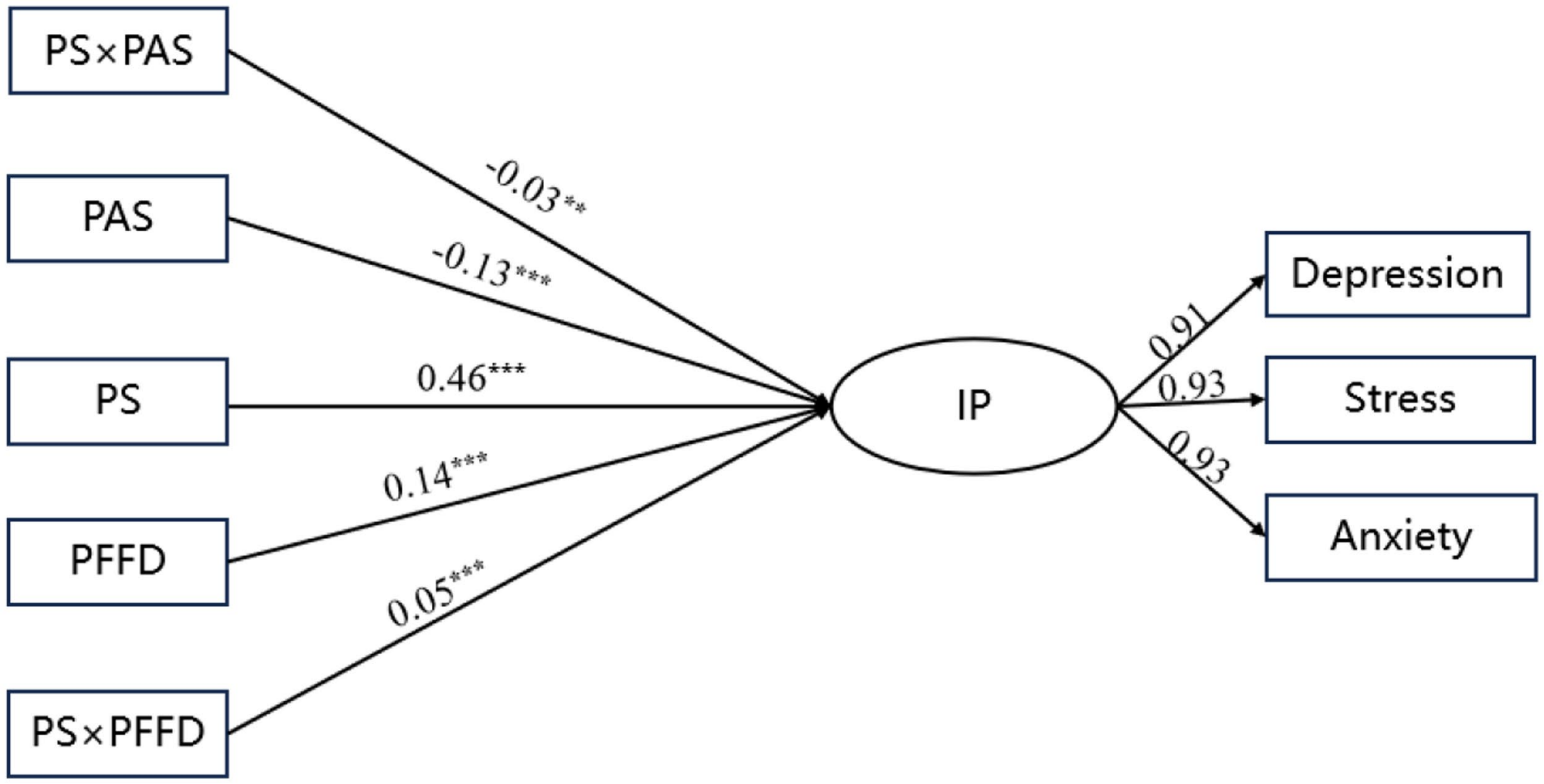
Structural equation model results of the overall sample. PAS, parental autonomy support; PFFD, perceived family financial difficulty; PS, Peer Stress; IP, Internalizing Problems. **p < 0.01, ***p < 0.001. For simplicity of the model graph, the predictive paths of the control variables are not shown.

Results for the total sample (Figure 2) showed that peer stress (β = 0.46, *p* < 0.01, 95% CI [0.438, 0.485]) and perceived family financial difficulty (β = 0.14, *p* < 0.01, 95% CI [0.121, 0.168,]) were positively associated with internalizing problems, whereas parental autonomy support (β = −0.13, *p* < 0.01, 95% CI [−0.152, −0.107]) was negatively associated with internalizing problems. The interaction between peer stress and parental autonomy support was statistically significant but small in magnitude (β = −0.03, *p* < 0.05, 95% CI [−0.053, −0.003]). Similarly, the interaction between peer stress and perceived family financial difficulty was statistically significant and small (β = 0.05, *p* < 0.01, 95% CI [0.023, 0.084]).

To examine developmental differences, multi-group comparisons of the moderation model were conducted across junior high school, senior high school, and university students. An unconstrained model was compared with constrained models in which the main and interaction effects of peer stress, parental autonomy support, and perceived family financial difficulty were set equal across paired groups. Group differences were evaluated using chi-square difference tests and the critical ratio for differences (CRD), with |CRD| > 1.96 indicating significant cross-group differences.

**Figure 3:**
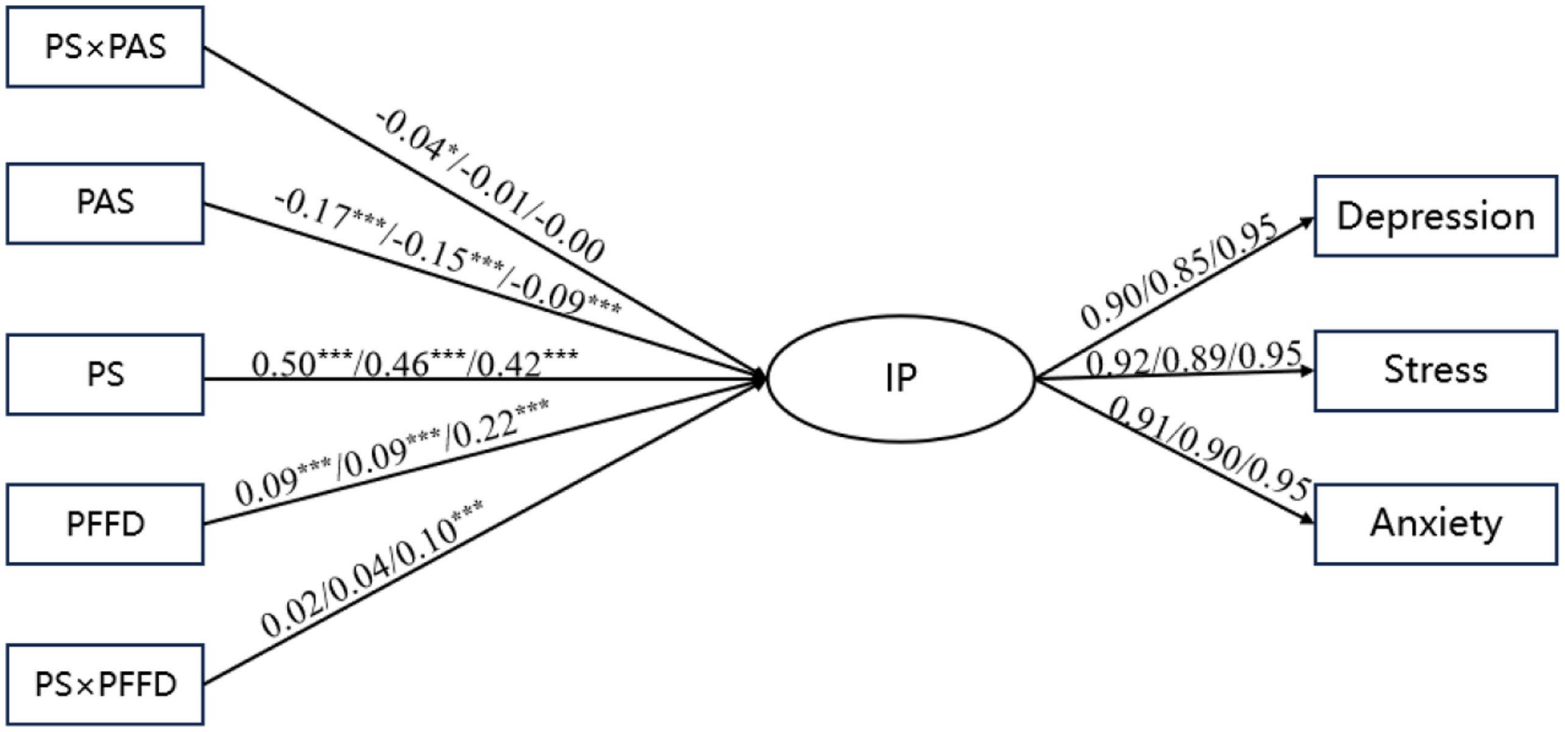
Educational stage group comparison results of modified structural equation modeling. Left coefficient/middle coefficient/right coefficient: junior high/senior high/university. *p < 0.05, ***p < 0.001.

As shown in Figure 3, the constrained models fit significantly worse than the unconstrained model for all comparisons (junior high vs. senior high: Δ*χ*^2^ = 31.05, Δdf = 5, *p* < 0.001; senior high vs. university: Δ*χ*^2^ = 91.21, Δdf = 5, *p* < 0.001; junior high vs. university: Δ*χ*^2^ = 55.87, Δdf = 5, *p* < 0.001), indicating significant stage differences in regression coefficients. Multi-group measurement invariance analyses were conducted for the latent internalizing construct. Constraining factor loadings to equality across groups (metric invariance) did not significantly worsen model fit (Δχ^2^(4) = 4.85, *p* = 0.303; ΔCFI < 0.01), supporting metric invariance. Therefore, structural path comparisons across educational stages were considered interpretable.

The effect of peer stress on internalizing problems was stronger among junior high school students (β = 0.50, *p* < 0.001, 95% CI [0.458, 0.541]) than senior high school students (β = 0.46, *p* < 0.001, 95% CI [0.406, 0.506]; |CRD| = 4.43) and university students (β = 0.42, *p* < 0.001, 95% CI [0.385, 0.458]; |CRD| = 4.99), whereas no difference was observed between senior high school and university students (|CRD| = 0.98 < 1.96).

The direct protective effect of parental autonomy support was stronger among junior high school students (β = −0.17, *p* < 0.001, 95% CI [−0.207, −0.133]) than university students (β = −0.09, *p* < 0.001, 95% CI [−0.122, −0.051]; |CRD| = 3.58) and stronger among senior high school students than university students (β = −0.15, *p* < 0.001, 95% CI [−0.192, −0.099]; |CRD| = 2.02), with no significant difference between junior high and senior high school students (|CRD| = 1.07 < 1.96). In contrast, the direct effect of perceived family financial difficulty on internalizing problems was stronger among university students (β = 0.22, *p* < 0.001, 95% CI [0.184, 0.254]) than both junior high school students (β = 0.09, *p* < 0.001, 95% CI [0.53, 0.126]; |CRD| = 5.03) and senior high school students (β = 0.09, *p* < 0.001, 95% CI [0.044, 0.147]; |CRD| = 5.47), with no significant difference between junior high school and high school students (|CRD| = 0.42 < 1.96).

Regarding the moderating effect, the interaction between peer stress and parental autonomy support was not significant in any of the three educational stages, with 95% confidence intervals including 0. In contrast, the moderating effect of perceived family financial difficulty differed across stages, being stronger among university students (β = 0.10, *p* < 0.001, 95% CI [0.053, 0.141]) than junior high school students (β = 0.02, *p* = 0.30 > 0.05, 95% CI [−0.017, 0.055]; |CRD| = 3.61) and senior high school students (β = 0.04, *p* = 0.79 > 0.05, 95% CI [−0.049, 0.119]; |CRD| = 2.99), while the difference between middle school and high school students was not significant (|CRD| = 0.53 < 1.96).

**Figure 4:**
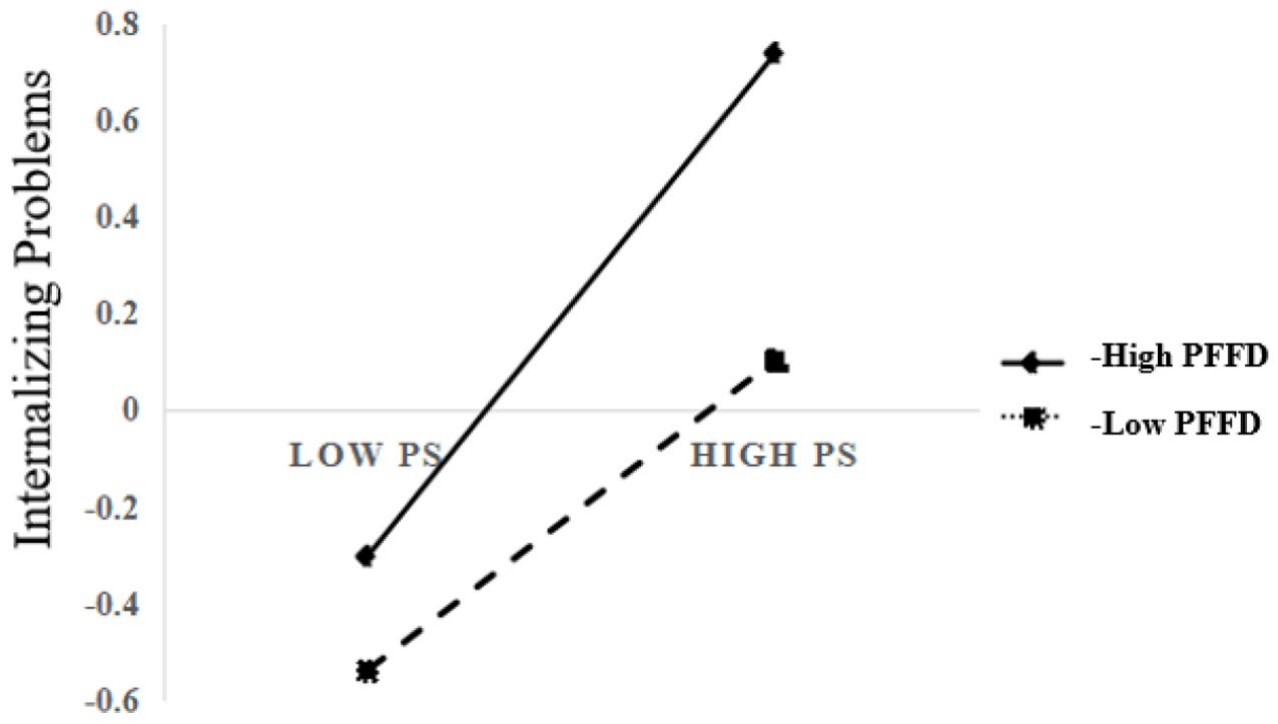
Interaction effect diagram. PFFD, Perceived Family Financial Difficulty; PS, Peer Stress.

Figure 4 presents the moderating effect of perceived family financial difficulty among college students. When perceived family financial difficulty was low, peer stress showed a weaker association with internalizing problems (β = 0.34, *p* < 0.01, 95% CI [0.290, 0.395]) than when perceived family financial difficulty was high (β = 0.50, *p* < 0.01, 95% CI [0.456, 0.544]).

## 4 Discussion

Based on a large-scale survey of adolescents in central China, this study systematically examined how peer stress, parental autonomy support, and perceived family financial difficulty jointly influence adolescents’ internalizing problems across educational stages. Within an integrated stress-buffering framework, the findings indicate that peer stress functions as a salient risk factor, whereas parental autonomy support and family economic conditions operate as contextual resources that shape adolescents’ psychological adjustment. Importantly, cross-sectional stage comparisons revealed that the strength and configuration of these associations differed across educational contexts.

### 4.1 Stage differences in main effects

Peer stress was a significant positive predictor of adolescents’ internalizing problems, such that greater exposure to peer aggression or exclusion was associated with higher levels of depression, anxiety, and stress. This finding is consistent with extensive prior research showing that peer bullying and exclusion exacerbate adolescents’ negative emotions and psychological distress across both Western and Chinese contexts (Chen et al., 2021; Christina et al., 2021; McClaine et al., 2024). In contrast, parental autonomy support was negatively associated with internalizing problems, indicating that supportive parenting serves protective role in adolescents’ mental health. This pattern aligns with developmental research emphasizing the importance of autonomy-supportive parenting in fostering emotional regulation and reducing emotional problems (Joussemet et al., 2008; Rueger et al., 2016). In contrast, perceived family financial difficulty was positively associated with internalizing problems, suggesting that subjective financial pressure represents a material vulnerability factor (Letourneau et al., 2013; Wadsworth et al., 2008). These main effects are consistent with conservation of resources theory, which posits that resource constraints heighten susceptibility to psychological distress (Hobfoll, 1989). Overall, these main-effect findings support the view that peer stress constitutes a risk factor for adolescent internalizing problems, whereas family support and material resources function as protective factors.

Cross-sectional comparisons revealed variation in the strength of the main associations across educational contexts, although the magnitude of these differences differed by predictor. First, peer stress was a robust predictor of internalizing problems across all educational stages. Although the coefficient was modestly higher in junior high school students (β = 0.50) than in senior high school (β = 0.46) and university students (β = 0.42), the absolute differences were small. Thus, the findings primarily indicate that peer stress exerts a consistently strong influence across educational contexts, with only limited stage-related variation. This pattern may suggest that peer stress is broadly central to adolescent emotional adjustment, while being slightly more salient in early secondary school settings. Early adolescence is often characterized by heightened concern with peer acceptance and social evaluation (Erath and Pettit, 2021), alongside still-developing social-cognitive and regulatory capacities (Laursen and Veenstra, 2021; Lerner, 2006). High school and university students tend to acquire more advanced social cognition, improved emotion regulation skills, and more diversified sources of self-identity, which may reduce their reliance on peer feedback and vulnerability to isolated peer stressors (Potterton et al., 2022; Vetter et al., 2013). However, given the modest magnitude of coefficient differences and the cross-sectional design, these interpretations should be viewed as contextual rather than developmental.

Conversely, more pronounced stage differences were observed in the main effects related to family. The negative association between parental autonomy support and internalizing problems was stronger among junior and senior high school students than among university students. This pattern is consistent with developmental systems and bioecological perspectives, which emphasize that adolescents’ embeddedness within family microsystems varies across contexts (Bronfenbrenner and Morris, 2006; Lerner, 2006). In middle and high school contexts, adolescencets’ daily proximal processes remain strongly family-centered, and autonomy-supportive parenting may directly shape emotional regulation capacities (Joussemet et al., 2008; Mcelhaney et al., 2009). In contrast, university contexts are characterized by increased autonomy expectations, physical separation from parents, and diversified identity domains (Arnett, 2000), which may attenuate the immediate salience of parental influence.

The most pronounced stage differentiation emerged for perceived family financial difficulty. The association between economic strain and internalizing problems was considerably stronger among university students (β = 0.22) than among junior and senior high school students (both β = 0.09). This finding aligns with evidence from studies of U. S. adolescents showing that the association between family economic status and mental health is stronger in later adolescence (Rivenbark et al., 2019, 2020). One possible explanation is that financial considerations become more directly salient in university environments, where students frequently encounter tuition costs, living expenses, and future career planning (Arnett, 2000; Jiang et al., 2021). Under such conditions, subjective perceptions of family economic constraints may play a more visible role in shaping emotional security and perceived life control (Jiang et al., 2021; Moore et al., 2021).

### 4.2 Moderation patterns

Overall, the moderation effects observed in the present study were relatively modest in magnitude. Although several interaction terms reached statistical significance, the coefficients were small. This indicates that despite its direct protective role—parental autonomy support showed limited ability to buffer the association between peer stress and internalizing problems. Suggesting that family-related factors primarily influenced adolescents’ internalizing problems through their direct associations rather than through strong buffering mechanisms. However, perceived family financial difficulty demonstrated variation in its moderating effect across educational contexts. The stress-amplifying effect of financial difficulty was strongest among university students, followed by senior high school students, and weakest among junior high school students. This pattern suggests that the contextual influence of financial strain on adolescents’ responses to peer stress may depend on the salience of economic considerations within particular educational environments. In university settings, where financial independence, tuition costs, and socioeconomic comparisons may become more visible, perceived economic strain may intensify the psychological impact of peer-related stressors (Arnett, 2000; Chen et al., 2015; Vosylis and Klimstra, 2022).

### 4.3 Implication

The present findings contribute to theoretical understanding of adolescent internalizing problems in several ways. First, the study reinforces the central role of peer stress as a core interpersonal risk factor for adolescents’ emotional distress. The robust association between peer victimization, exclusion, and internalizing symptoms observed across educational contexts supports interpersonal stress models of psychopathology and highlights the importance of peer-related stressors in adolescent mental health (Erath and Pettit, 2021). Second, the findings support the role of parental autonomy support as a psychological family resource associated with adolescents’ emotional well-being. The protective association between autonomy-supportive parenting and internalizing problems was most pronounced among junior high school students, suggesting that psychological family resources may be particularly salient in educational contexts where adolescents remain more closely embedded in family processes. Third, the results extend resource-based perspectives on adolescent stress by highlighting the contextual role of material resource constraints. Perceived family financial difficulty showed a stronger association with internalizing problems among university students and demonstrated a modest amplification effect on the association between peer stress and psychological distress in this context. This pattern suggests that material resource constraints may become more salient in environments where financial demands and socioeconomic comparisons are more visible. Taken together, these findings highlight the importance of distinguishing between psychological and material family-related domains within the stress-buffering framework (Cohen and Wills, 1985). Moreover, the differing results observed across educational contexts suggest that the influence of distinct resource domains on internalizing problems and stress responses may vary in contextual salience.

These findings provide empirical support for implementing stage-specific and targeted mental health prevention and intervention strategies. For junior and senior high school students, interventions should adopt a dual focus: strengthening adolescents’ social skills and resilience to cope with peer stress, while also enhancing parent education programs that promote autonomy-supportive parenting and equip parents to better support their children’s emotional well-being. For university students, mental health services should adopt a stronger socioeconomic perspective. In addition to routine counseling, greater emphasis should be placed on systematic support for economically disadvantaged students, including comprehensive scholarship and financial aid programs, accessible low-cost mental health services, and the promotion of a campus culture that discourages conspicuous consumption. Such efforts may help reduce the adverse mental health effects associated with economic disparities.

### 4.4 Limitation

Several limitations warrant consideration. First, the cross-sectional design does not permit inferences about within-individual developmental change. The observed differences across educational stages represent between-group contrasts rather than longitudinal developmental processes. Future longitudinal or cohort-sequential studies are needed to clarify how stress–resource dynamics unfold across adolescence and emerging adulthood. Second, all measures were self-reported, which may introduce common method variance. Although preliminary tests did not indicate severe bias, common method variance may still inflate associations among variables. Multi-informant or objective indicators would strengthen future research. Third, the study focused on peer stress and family-related domains, leaving other developmentally salient systems unexamined. Incorporating additional ecological layers may further clarify age-graded risk and protective processes. Finally, these findings are embedded in the sociocultural context of central China and may not generalize to settings with different cultural norms or welfare structures. Future cross-cultural and cross-regional research is needed to examine the generalizability of the present findings. Taken together, these limitations highlight the need for longitudinal, multi-method research to further elucidate the risk and protective processes in adolescent mental health.

## 5 Conclusion

In summary, the present study examined how peer stress, parental autonomy support, and perceived family financial difficulty relate to adolescents’ internalizing problems across different educational contexts. Peer stress emerged as a consistent and robust predictor of internalizing symptoms across all groups. Parental autonomy support showed a protective association with adolescents’ emotional well-being, with the strongest effects observed among junior high school students. In contrast, perceived family financial difficulty demonstrated a stronger association with internalizing problems among university students and showed a modest amplification effect on the association between peer stress and psychological distress in this context. Taken together, these findings emphasize the central role of peer stress in adolescents’ internalizing problems. And they also highlight the importance of distinguishing between psychological and material family-related domains within stress-buffering frameworks. By jointly examining these domains, the present study provides a more nuanced theoretical perspective on how family-related resources shape adolescents’ responses to peer stress.

## Data Availability

The raw data supporting the conclusions of this article will be made available by the authors, without undue reservation.
